# Supplementary material for: Cortical and Subcortical Brain Volumes Partially Mediate the Association between Dietary Composition and Behavioral Disinhibition: A UK Biobank Study
Source: Nutrients. 2021 Oct 9;13(10):3542. doi: 10.3390/nu13103542 (PMC8537365; doi:10.3390/nu13103542)
Supplement: Supplementary file 1 [file nutrients-13-03542-s001.zip › nutrients-1354604-SI.pdf]

Supplementary methods

Below several supplemental tables show the composition of the dietary components based on the FFQ items, as well as the composition of the Disinhibition score based on assorted impulsivity related items and information.

Table S1. UK Biobank items related to disinhibition, impulsivity, compulsivity and/or emotional instability and their binarised response categories.

Data-field	Source	Description	Response categories (original)	Response categories (binary)
1239	IAV	Do you smoke tobacco now?	- No<br>- Yes, on all/most days<br>- Only occasionally	- No / only occasionally<br>- Yes, on all/most days
1920	IAV	Does your mood often go up and down?	- No<br>- Yes	- No<br>- Yes
1940	IAV	Are you an irritable person?	- No<br>- Yes	- No<br>- Yes
1960	IAV	Do you often feel fed-up?	- No<br>- Yes	- No<br>- Yes
20544	MHQ	Have you been diagnosed with one or more of the following mental health problems by a professional, even if you don't have it currently? (tick box)	- OCD<br>- Mania<br>- ADHD	- No<br>- Yes<br>(per diagnosis)
20548	MHQ	Try to remember a period when you were in a "high" or "irritable" state (tick box) (conditional to: ever had period of extreme irritability/excitability)	- More restless than usual<br>- Thoughts were racing<br>- Easily distracted<br>- More active than usual	- No<br>- Yes, ever had period of mania/irritability with at least one of these symptoms
20401	MHQ	Have you been addicted to or dependent on one or more things, including substances (not	- No<br>- Yes	- No<br>- Yes

		cigarettes/coffee) or behaviours (such as gambling)?		
20416	MHQ	How often do you have six or more drinks on one occasion? (conditional to: frequency of drinking alcohol)	- Never<br>- Less than monthly<br>- Monthly<br>- Weekly<br>- (Almost) daily	- Never / less than weekly<br>- Weekly / (almost) daily
20453	MHQ	Have you taken cannabis (marijuana, grass etc.), even if it was a long time ago?	- No<br>- Yes, 1-2 times<br>- Yes, 3-10 times<br>- Yes, 11-100 times<br>- Yes, more than 100 times	- No / less than 11 times<br>- Yes, 11 times / more
20480	MHQ	Have you deliberately harmed yourself, whether or not you meant to end your life?	- No<br>- Yes	- No<br>- Yes
41202 and 41204	LHR	Main or secondary ICD-10 diagnoses from hospital inpatient records	See supplementary table ST1.2	- No<br>- Yes<br>(per diagnostic group)

Abbreviations: IAV= initial assessment visit, MHQ= mental health questionnaire, LHR= linked health records, ICD-10= international classification of diagnoses, 10th edition.

Table S2. ICD-10 diagnoses related to impulsivity, compulsivity and/or emotional instability per diagnostic group.

Addiction	F102	Dependence syndrome of alcohol
	F112	Dependence syndrome of opioids
	F122	Dependence syndrome of cannabinoids
	F132	Dependence syndrome of sedatives or hypnotics
	F142	Dependence syndrome of cocaine
	F152	Dependence syndrome of other stimulants
	F162	Dependence syndrome of hallucinogens
	F192	Dependence syndrome of multiple drug use and use of other psychoactive substances
Externalising	F900	Disturbance of activity and attention

	F901	Hyperkinetic conduct disorder
	F908	Other hyperkinetic disorders
	F909	Hyperkinetic disorder unspecified
	F910	Conduct disorder confined to the family context
	F911	Unsocialised conduct disorder
	F912	Socialised conduct disorder
	F913	Oppositional defiant disorder
	F918	Other conduct disorders
	F919	Conduct disorder unspecified
	F920	Depressive conduct disorder
	F928	Other mixed disorders of conduct and emotions
	F929	Mixed disorder of conduct and emotions unspecified
Mania	F300	Hypomania
	F301	Mania without psychotic symptoms
	F302	Mania with psychotic symptoms
	F308	Other manic episodes
	F309	Manic episode unspecified
	F310	Bipolar affective disorder current episode hypomanic
	F311	Bipolar affective disorder current episode manic without psychotic symptoms
	F312	Bipolar affective disorder current episode manic with psychotic symptoms
	F313	Bipolar affective disorder current episode mild or moderate depression
	F314	Bipolar affective disorder current episode severe depression w/o psychotic symptoms
	F315	Bipolar affective disorder current episode severe depression with psychotic symptoms
	F316	Bipolar affective disorder current episode mixed
	F317	Bipolar affective disorder currently in remission
	F318	Other bipolar affective disorders
	F319	Bipolar affective disorder unspecified
OCD	F420	Predominantly obsessional thoughts or ruminations
	F421	Predominantly compulsive acts [obsessional rituals]
	F422	Mixed obsessional thoughts and acts
	F428	Other obsessive-compulsive disorders

	F429	Obsessive-compulsive disorder unspecified
	F633	Trichotillomania
	F950	Transient tic disorder
	F951	Chronic motor or vocal tic disorder
	F952	Combined vocal and multiple motor tic disorder [de la Tourette]
	F958	Other tic disorders
	F959	Tic disorder unspecified
	F605	Anankastic personality disorder
Personality	F603	Emotionally unstable personality disorder
Risk-taking	F100	Acute intoxication with alcohol
	F101	Harmful use of acohol
	F110	Acute intoxication of opioids
	F111	Harmful use of opioids
	F120	Acute intoxication cannabinoids
	F121	Harmful use cannabinoids
	F130	Acute intoxication of sedatives or hypnotics
	F131	Harmful use of sedatives or hypnotics
	F140	Acute intoxication of cocaine
	F141	Harmful use of cocaine
	F150	Acute intoxicationof other stimulants
	F151	Harmful use of other stimulants
	F160	Acute intoxication of hallucinogens
	F161	Harmful use of hallucinogens
	F190	Acute intoxication due to multiple drug use and use of other psychoactive substances
	F191	Harmful use due to multiple drug use and use of other psychoactive substances
	F630	Pathological gambling
	F631	Pathological fire-setting [pyromania]
	F632	Pathological stealing [kleptomania]
	F638	Other habit and impulse disorders
	F639	Habit and impulse disorder unspecified

Table S3: Cases per behavioural group, its sensitivity and factor loading.

Group	Loading	Definition
Externalising	0.589	a) participant self-reports diagnosis of ADHD by professional – OR –
		b) hospital diagnosis of any externalising disorder
OCD	0.591	a) participant self-reports diagnosis of OCD by professional – OR –
		b) hospital diagnosis of OCD/tic disorder/trichotillomania/anankastic PD
Addiction	0.708	a) participant self-reports past or current addiction – OR –
		b) hospital diagnosis of substance dependence syndrome
Self-harm	0.637	a) participant self-reports past or current self-harm
Personality	0.536	a_1) participant self-reports moods going up and down – AND –
		a_2) participant self-reports being easily fed-up – AND –
		a_3) participant self-reports being irritable – OR –
		b) hospital diagnosis of emotionally unstable PD
Mania	0.613	a) participant self-reports diagnosis of mania by professional – OR –
		b) hospital diagnosis of mania/bipolar disorder – OR –
		c) participant has experienced period with at least one symptom of mania
Risk-taking	0.335	a) participant self-reports weekly binge-drinking – OR –
		b) hospital diagnosis of acute intoxication with alcohol/drugs
Cannabis	0.552	a) participant self-reports having used cannabis >10 times in life
Smoking	0.475	a) participant self-reports current daily smoking

Abbreviations: ADHD = attention-deficit/hyperactivity disorder; OCD = obsessive-compulsive disorder; PD = personality disorder

Table S4. UK Biobank items related to diet. Note that items related to alcohol intake are not included, since heavy drinking contributed to our outcome of interest.

**Type**	**UKB ID**	**Item**	**Unit**
Continuous	1289	Cooked vegetables	Tablespoons / day
	1299	Raw vegetables	Tablespoons / day
	1309	Fresh fruit	Pieces / day
	1319	Dried fruit	Pieces / day
	1438	Bread	Slices / week
	1458	Cereal	Bowls / week
	1488	Tea	Cups / day
	1498	Coffee	Cups / day
	1528	Water	Glasses / day
Frequency	1329	Oily fish	Times / week
	1339	Non-oily fish	Times / week
	1349	Processed meat	Times / week
	1359	Poultry	Times / week
	1369	Beef	Times / week
	1379	Lamb	Times / week
	1389	Pork	Times / week
	1408	Cheese	Times / week
	1478	Added salt	Never or rarely / sometimes / usually / always
Type	1418	Milk	Full-cream / semi-skimmed / skimmed / soy / other non-dairy / never

	1428, 2654	Spreads	Butter / block margarine / tub margarine / benecol / olive-oil based / sunflower-based / low-fat / other / never
	1468	Cereal	Bran / biscuit / oat / muesli / refined sugar-sweetened / never
	1508	Coffee	Decaffeinated / instant / ground / other / never
	1448	Bread	Brown / white / wholegrain / other / never
Elimination	6144	I never eat (tick box)	Eggs / dairy / wheat / sugar

Table S5. Factor loadings (≤-0.20 or ≥0.20) per item for each dietary component (DC1-4)

	**DC1**	**DC2**	**DC3**	**DC4**
Wholegrain bread vs. any other bread	0.59			
Dried fruit frequency	0.48			
Oily fish frequency	0.47		0.27	
Raw vegetables / salad frequency	0.45			
Fresh fruit frequency	0.45			
Cooked vegetables frequency	0.37			
Water frequency	0.34			
Fish frequency	0.33		0.33	
Cereal frequency	0.32			
Ground coffee vs. any other coffee	0.32			
Cereal vs. 'I never eat cereal'	0.29	-0.22		
Muesli vs. any other cereal	0.26			
Processed meat frequency	-0.24		0.48	
Instant coffee vs. any other coffee	-0.32			
Refined sugar-sweetened cereal products	-0.33			
White bread vs. any other bread	-0.56			
'I never eat dairy' vs. no restrictions		0.64		
'I never eat wheat' vs. no restrictions		0.64		
I never eat eggs, sugar, wheat or dairy' vs. no restrictions		0.52		
'I never eat eggs' vs. no restrictions		0.50		

‘I never eat dairy’ vs. no dairy restrictions	0.33		
‘I never eat wheat’ vs. no wheat restrictions	0.33		
Soy milk vs. any other milk	0.27		
‘I never eat eggs’ vs. no eggs restrictions	0.23		
Other bread vs. any other bread	0.21		
Other milk vs. any other milk	0.20		
Coffee vs. ‘I never drink coffee’	-0.20		
Milk vs. ‘I never drink milk’	-0.23		0.27
Bread frequency	-0.26		
‘I never eat sugar’ vs. no restrictions	-0.35		
Bread vs. ‘I never eat bread’	-0.38		
Lamb frequency		0.75	
Beef frequency		0.73	
Pork frequency		0.73	
Poultry frequency		0.40	
Added salt frequency		0.31	
Fat content of milk			0.83
Semi-skimmed milk vs. any other milk			0.55
Full cream milk vs. any other milk			0.30
Butter vs. any other spread			0.28
Cheese frequency			0.25
Skimmed milk vs. any other milk			-0.84
